# On the Treatment of Chronic Hydrocephalus

**Published:** 1859-04

**Authors:** Daniel Brainard

**Affiliations:** Professor of Surgery in Rush Medical College


THE CHICAGO

### MEDICAL JOURNAL.

VOL. II. APRIL, 1859. No. 4.

### ORIGINAL COMMUNICATIONS.

ARTICLE I.

### ON THE TREATMENT OF CHRONIC HYDROCEPHALUS BY INJECTIONS OF IODINE.

BY DANIEL BRAINARD, M.D., PROFESSOR OF SURGERY IN RUSH MEDICAL COLLEGE.

In the winter of 1850, I published the report of a case of hydrocephalus, treated by iodine injections, which, although it eventually terminated fatally in the usual way, was yet sufficient to demonstrate the harmlessness of the treatment employed, and its value as a palliative. Since that time, another case has been treated by the same method with success.

These, it would seem, ought to be sufficient to remove all doubts concerning the propriety of resorting to this method of treatment in favorable cases. Still so large a proportion of the profession continue to regard it as a curious experiment, or a rash and dangerous operation, and in either case unjustifiable, that I have thought it best to group together the reasons which induced me to venture upon, and afterwards recommend it. These reasons are founded in analogy, the nature of the disease, the insufficiency of other means of treatment, and the peculiar applicability of this method to this form of disease.

1. *Analogy.*

Experience has shown, that injections of iodine are uniformly successful in two forms of cyst, viz: where the contents are serous, and where the surfacc is not too large; hence, in hydrocele, serous cysts of the neck, and spina bifida they have very uniformly succeeded, while in cutaneous, albuminous and sanguine cysts they have often failed, and in ascites they have proved dangerous, on account of the extent of the surface affected. In these respects, chronic hydrocephalus enters into the most favorable class; it is more uniformly serous than any other dropsical effusion, and it is confined in a cyst of limited extent, scarcely exceeding that of hydrocele.

2. *The nature of the disease.*

Chronic hydrocephalus is an encysted dropsy, in which a greater quantity of serum, than natural, exists in the ventricles of the brain, and it is, therefore, not to be regarded as simply an abnormal increase of the cerebro-spinal fluid.

Todd and Bowman, a very competent authority on this subject, are of opinion that it is distinct from it. "This fluid, which fills the sub-arachnoid spaces during life, keeps the opposed surfaces of the arachnoid membrane in contact." "The ventricles of the brain contain a secretion of very similar, if not identical characters, which Magendie describes as communicating with that of the sub-arachnoid space through an orifice in the inferior extremity of the fourth ventricle. This, however, is extremely doubtful, as the fluid of the ventricles is enclosed by a membrane which lines their cavity."*

Concerning this membrane, Kollicker expresses himself as follows: "All the portions of the ventricles, which are not lined by the continuation of the pia mater, have a special lining membrane, the so-termed *ependyma ventriculorum.*" "This is a simple tessellated epithelium. It lies normally immediately upon the nerve substance, but there is so frequently developed beneath it a filamentous layer, resembling connective tissue

* Physiological Anatomy of Man, p. 231.

(fibrous tissue) 0.01–0.05 of a line thick, that its occurrence at a certain age might almost be described as constant, as in fact it is by Virchow."*

No nerves have been as yet discovered in the membranes of the ventricles. The plexus choroides is composed of white fibrous tissue and blood vessels. The arachnoid, according to Kollicker, has no proper vessels. These facts are only referred to here as leading to a correct understanding of the manner in which the sac is formed in hydrocephalus, and as showing the entire absence of sensibility and irritability in all the parts of the surface concerned in the formation of the lateral ventricles.

Fleurens† found that the cerebrum, including the corpora quadrigemina, the optic thalami and the corpora striata, might be pricked and lacerated without giving rise to any sign of suffering or convulsive movements, and the same results have been obtained by other experimenters.

The fibrous layer of the *ependyma* ventriculorum is constantly found thickened in hydrocephalus (Kollicker op. cit.) and "brain sand" and *corpuscula amylacea* are often found in the walls of the ventricles.

"The general anatomical characters of chronic hydrocephalus are a large accumulation in the ventricles of a clear and colorless serum, which contains very little animal matter, and a thickening and toughening of their lining membrane, for the most part, to a considerable degree."‡ "I believe the really essential part of congenital hydrocephalus is the affection of the *ependyma*."§

The view that chronic hydrocephalus is a disease of the membrane lining the ventricles confined to these cavities for the most part, and not communicating with the cavity of the arachnoid (so called), or with the sub-arachnoid space, is important, as bearing on the question of treatment. Without, therefore, going into a discussion of the various forms which the disease presents, I shall add such proofs of this view, drawn from recent and reliable sources, as will leave no doubt upon the subject.

---

* Manual of Human Microscopical Anatomy, p. 376 et seq.

† Du System Nerveux, p. 19.

‡ Rokitansky Path. Anat., vol. 3, p. 274. § Ib. p. 276.

M. Blache, in a memoir, presented to the Academy of Medicine, at its sitting of May 29, 1855, arrives at the following conclusions, the result of extensive researches.

1st. " In chronic hydrocephalus of the ventricles, the fluid accumulates in the lateral ventricles, rarely in the fourth ventricle. It does not communicate with the periphery of the brain, nor with the spinal cord.

5th. " The internal membrane of the ventricles becomes so thick that it may be dissected at all points, showing that it is continuous throughout and can have no communication except by the aqueduct of Sylvius.

8th. In two cases, we have not been able to find the aqueduct of Sylvius, and if we are not deceived, the cerebral cavities were completely closed.

9th. Nothing in the walls, nor in the liquid effused, indicated inflammation. We consider it a dropsy *pure et simple*."*

Riellet and Barthez regard chronic hydrocephalus as a " non-inflammatory effusion into the cranial cavities." They consider the acute form of the disease as "a mere epiphenomenon of tubercular meningitis."

The alteration which the *ependyma* undergoes in chronic hydrocephalus is not always the same. In a case reported by Dr. Dugas, it was found on dissection, that " the inner surface of the *cerebral sac* resembled very closely a healthy mucous coat of the stomach. In this case flakes of a substance of a whitish color, some of which resembled pus, were found at different points. *

In a case which I examined, the whole internal surface of the ventricles was much firmer than natural, and the choroid plexus much enlarged, covered with granulations and very vascular, showing numerous small serous cysts in its substance. The orifice of the duct of Sylvius could not be discovered. When this canal remains pervious in chronic hydrocephalus, the fourth ventricle is dilated, as in the cases related by Klein and others.‡ In this case, which is reported further on, and which had been

* Gazette Medicale, for 1855, p. 346.
† American Journal of the Medical Sciences, vol. 20, p. 536.
‡ Dict. de Medicine, vol. 15.

treated by injections of iodine, there did not seem to be any lining membrane remaining, or it was so fine and attenuated as to be imperceptible. The fluid in this case seemed to have been secreted by the choroid plexus, and it is probable that it is to this circumstance, which seems unusual, that the failure of the treatment is to be attributed.

3. *Other treatment generally unsuccessful.*

No doubt can exist concerning the generally unsuccessful result of tapping in chronic hydrocephalus. The table published in the *Medical Gazette*, by Dr. Charles West, and republished by Professor Blackman, with additions in the *N. Y. Journal of Medicine* for May, 1854, seems to show that of sixty-nine cases in which it has been used, in nineteen the patients survived for four months. This is no doubt a much more favorable result than would be presented could all the cases which have been operated upon be collected, and it would be by no means difficult to add cases and authorities adverse to the practice. Still as a sole resort in a generally hopeless disease its performance is justifiable.

There can, however, be no doubt that tapping does sometimes effect a cure. Sanc Vosa, Halibrouck, Syme, Chassaignac, Adams, Bedor, Graefe, Crampton, Monod, Conquest and others, have reported cases of the kind, and if we admit that in some of these the cure may not have been perfect and permanent, this cannot reasonably be presumed to have been the case in regard to all. The operations by puncture show that while the practice is insufficient, the disease is not in its nature incurable.

The table of Dr. West, however carefully examined, throws but little light on the question of tapping. In most of the cases neither the appearance of the lining membrane of the ventricles, nor the color and consistence of the fluid, points essential to the correct understanding of the disease, is given. In two of the cases reported cured, it is stated that the fluid was clear and limpid at the first puncture, and became afterwards thicker and turbid, indicating that it was the inflammation excited in the lining membrane of the ventricles, and not the mere discharge of fluid, which effected a cure.

It is also noticeable that the cases in which punctures were made with the couching needle, and in which but a small quantity of fluid was evacuated, resulted more favorrbly than those in which a trochar was employed. Græfe and Crampton both used fine needles with success.

*Compression.*

It is probable that a tabular view of the cases treated by compression would present a more favorable result than tapping. It has generally been resorted to in the earlier stages of the disease, and in the mildest form of it, and can at best be regarded only as a palliative.

When we consider the effects of pressure on the cerebral substance, it must be obvious that this treatment can only be applicable to the degree of affording to the thin and imperfectly formed coverings of the brain that power of supporting the brain which they have lost. In many cases, where the bones of the cranium are joined, patients die from the effects of pressure.

The principle of treating hydrocephalus must be the same as that of spina bifida hydrocele and serous cysts. It is beyond the reach of medicine administered by the mouth, and of external applications. Simply removing the fluid by tapping has, as in other dropsies, little tendency to cure it. But the exciting upon the internal surface of the lining membrane of the ventricles a moderate degree of inflammatory action changes the structure of the membrane, and alters the character of the fluid secreted, and, as in hydrocele, must cure the disease.

To effect this result, it is only necessary to excite a very low degree of inflammatory action. I have cured a case of ancient hydrocele by two successive injections of one fluid drachm of a solution of iodine, containing one grain to three grains of iodide of potassium. This was thrown in through the canula of a fine exploring trochar without evacuating the fluid. The patient, a laborer, continued his work during the treatment, and only felt a very slight degree of tenderness in the tumor, which at the end of four weeks was entirely removed. Other examples of the removal of the serous contents of cysts by the same means might be cited. According to the laws of *endosmosis* and *exosmosis*, we are taught that it is in many cases only necessary to change in a slight degree the constitution of a fluid, to cause it to be absorbed rapidly. How far the same law may be applicable to membranes in the living body it is impossible to say, but there can be no doubt that, that change in structure of tissue, produced by a slight degree of inflammation, is sufficient to permanently affect its action of exhalation and absorption. This, according to the present knowledge, can most safely be effected by injections of iodine. But, here we are met by the objection, that exciting such inflammation is too dangerous. I have already given reasons sufficient for believing that such is not the case. The absence of sensibility, of irritability, of nerves, and red vessels in the *ependyma*, all indicate the contrary. The fact that the fluid in some cases of cure by tapping became fibrinous before absorption commenced, coincides with them. But to confirm these indications, I shall add two cases of idiopathic inflammation of the serous membrane lining the ventricles; one occurring in a healthy state, and resulting fatally; the other occurring during hydrocephalus, and resulting in a cure, in order to demonstrate the very great extent to which inflammatory action so situated may be carried before it proves fatal, and even without proving fatal. The first case is quoted from an article by Riellet, in the *Archives Generales de Medicine* for Dec., 1847:

CASE 1. "An intelligent and well developed young girl, aged ten years and one-half, was seized suddenly with an acute headache, with vomiting, fever, and dread of light and sounds. After four days she had an attack of convulsions. The seventh day there was some difficulty of hearing. The vomiting, pain, and convulsions continued. The twenty-first day there was complete deafness. The fifty-second day the intelligence diminished, and the convulsions were less frequent and severe.

"The patient died at the end of four months, from a renewed attack of convulsions.

"The principal appearances found on dissection were the presence of ten ounces of perfectly limpid serum in the lateral ventricles, which coagulated 'like the white of an egg,' on the application of heat.

"The ventricular membrane, throughout its entire extent, offered a gelatinous appearance, was a *milimetre* in thickness resembled the mucous coat of the stomach where affected with the commencement of gelatinous softening; but far from being softened, this was more consistent than natural, and could be detached throughout its whole extent. The cerebral substance was throughout of good consistence, not at all injected."

In this case, the most acute inflammation of the ventricular membrane, continued for four months, had produced no organic disease not consistent with life, if the fluid had been drawn off, and it is not a little singular that tapping should not be more frequently thought of in the acute form of the disease, in which it seems more urgently indicated than in the chronic.

Case 2.—Dr. Conquest, in April, 1829 or 1830, published a case of tapping in hydrocephalus:—

"This child, a girl of about two years of age, had several signs of hydrocephalus, from a date soon after birth, and for many months past, the head gradually increased until it acquired an enormous size. The forehead was unusually broad, and the anterior fontanelle unusually large. The pupils were permanently dilated. The child slept almost constantly, and frequently had two or three frightful convulsions during the day and night."

Dr. Conquest operated, introducing the trochar into the right lateral ventricle, an ounce and a half of bloody serum, mixed with portions of cerebrum, escaped. The fluid was allowed to escape stillicidium, and within forty-eight hours about two pints and a half flowed out of the opening.

Some symptoms of inflammation of the brain followed, which required leeching.

"When this interesting child was exhibited to the class on Saturday evening, every one was struck with the improvement of its appearance, and by the intelligence and cheerfulness of its countenance. Dr. C. stated, that he considered it perfectly well."*

In this case, acute inflammation supervened upon chronic disease of the ventricular membrane, and was carried to the extent of producing softening, if not gangrene of the brain. Yet

* American Journal for 1830, p. 515, from London Medical Gazette.

we see that a timely evacuation of the serum of the ventricles was followed by a cure.

I say cure, for although some have denied the possibility of such a result in hydrocephalus, yet it can scarcely be doubted, that in this case the cure was as perfect as is often obtained in acute diseases, when some traces of the morbid condition remain through life.

If it be objected that this is an exceptional case, I reply, that such a degree of inflammation is by no means contemplated as a part of the treatment of hydrocephalus. It is only adduced as evidence of the extent to which inflammation of the ventricular lining may be carried *in* hydrocephalus without permanently injurious effects.

4. *Hydrocephalus treated by injection of iodine.*

CASE 1.—Dr. Tournesko, surgeon of the Civil Hospital of Koltsa, Bucharest, has recently communicated to the *Gazette des Hopitaux* a case of hydrocephalus, treated by injection of the tincture of iodine.

The subject was a child, two months old, and the head measured about twenty inches in circumference. At the first puncture, eleven ounces of serum were drawn. This was replaced by effusion in twenty-four hours. Another puncture was made the second day, and twenty-four ounces of fluid drawn off. This time the tincture of iodine was injected (twelve *grammes* of tr. of iodine in twenty-four *grammes* distilled water), of which one-eighth part was allowed to flow out. The quantity inserted contained, according to our calculation, about sixteen grains of iodine and three teaspoonsful of alcohol.

At the moment of the injection, the child turned pale and cried several times. The following day he had fever, for which calomel was prescribed.

Twenty days after the operation, the head was of the natural size, and the child was in good health. Thirty-five days after the operation, the child continued in the same satisfactory state.

The following are the details of the operation:

1. A large-sized trochar was used.
2. It was carried to the depth of about two inches.

3. The puncture was made in the coronal suture, at the side (as being the point nearest the ventricles), and at an angle of forty-five degrees with the horizon.

CASE 2.—Case treated by Dr. Brainard, from *N. W. Medical Journal*, 1850.

Sept. 20, 1849. Saw a female child of Mr. R. aged 4 days, affected with cleft spine and hydrocephalus.

The cleft of the spine was situated at the lower part of the lumbar region, was about 3 inches in length and 1½ in breadth; the abdomen was prominent, particularly on each side, the divisions of the spinal column movable, showing that the want of union was not confined to the processes, but extended to the bodies of the vertebræ.

There was a fluctuating tumor situated over the fissure, about three-fourths of an inch above the surface of the surrounding skin. Its covering was then of a blueish color, semi-transparent, and seemed composed of the thin membranes of the spinal cord, and the cuticle alone. Indeed, the latter was wanting over a considerable surface, where an ulceration of the size of a shilling existed.

The head was of large size, measuring 19 inches around the frontal and parietal protuberances. The upper part of the parietal and occipital bones was wanting, and the head bulged out into a very irregular, tense, and perfectly elastic sac. The other bones were imperfectly ossified and separated. The head was hot, covered with large veins, and increased rapidly in size. The right foot was affected with a talipes varus, and the movement of the lower extremities was feeble.

The urine dribbled away, and excoriated the skin extensively.

Applications of nit. arg., in solution, were directed for the ulcerated spot, until it was nearly healed. Cold water was applied to the head to keep down the heat.

Oct. 9, at 12½ o'clock, the treatment was commenced as follows:

A very minute puncture was made with the point of the lancet, through the skin, half an inch below the tumor of the back, and a small sized exploring trochar passed from it obliquely upward into the sac. Without allowing the escape of more than a few drops of fluid, f. dr. ss. of a solution of iodine, of the following strength, was injected: iodine, gr. j., iod. pot. gr. ij.; aq. dist. f. oz. j.; making 1-16 gr. of iodine and $\frac{1}{8}$ gr. of hyd. potas. in the injection. The puncture and surface of the tumor were then covered with a solution of gun cotton. No pain was felt at the moment of puncturing the skin, and from the application of the liquid plaster. I was assisted by Professors Evans and Herrick, and Dr. Meek, editor of the *N. W. Journal of Medicine.*

10th. 12 o'clock M. Found the child had been restless from the time of the operation until 6 o'clock P.M., when it became quiet; seems well. Veins of head less turgid than since its birth.

11th. Child appears well; head less tense. Applied adhesive liquid to tumor and continued cold water to head.

12th. Head tense; increased in size $\frac{1}{2}$ inch. Tumor of back flaccid, flattened and slightly inflamed.

13th. Tumor of back diminishing; head increasing rapidly. Ulceration of back cicatrized.

In consequence of the rapid increase in the size of the head, I determined to inject it without waiting, as I had intended to do, for the cure of the back to be completed. This was accordingly done with the assistance of Drs. Evans and Rutter. The point chosen was that most projecting on the summit and left side of the head.

About $\frac{1}{2}$ dr. of fluid was allowed to escape. The same quantity of the solution (of the strength before given) injected, and the puncture covered with adhesive solution. Child seemed to suffer no pain except from puncture and application of ether upon the point.

14th, 15th, 16th. Head hotter, and increased in size; appears well.

16th; 8 o'clock P. M. The puncture of head commenced leaking, which was stopped by Dr. Herrick with collodion.

17th, at 4 o'clock A. M., leaked again, and was stopped by a compress and bandage. Head flaccid; child irritable, sleepless, and starting.

19th. Head filling. To avoid danger of leaking from former puncture, 1½ oz. of fluid was drawn off by a very oblique puncture on the right side.

20th. Child restless; affected with apthæ. Head hot.

21st. Child appears well; head natural.

31st. Head, which was stationary, has commenced to increase; measures 20 inches in circumference. It was injected at 5 o'clock P. M., with f. dr. jss. of the solution. The puncture was made obliquely upon the summit of the right side.

Nov. 1st. For 24 hours no effect was perceptible. At the end of this time, it commenced to be restless and sleepless, twitching often, and declined the breast. Evaporating lotions were applied to the head; a cathartic of castor oil administered.

Nov. 2d. The child was much relieved, and took the breast. Head stationary; the whole of the back part of it covered with a red blush, which had gradually spread from the puncture.

Nov. 3d. Child restless. Head hot and red at the back part.

Nov. 4th. Heat less; redness almost gone; head diminished in circumference, ¾ inch in 24 hours.

5th. Child restless; head flaccid, diminished in size. Applied a band of India rubber, 4 inches broad, about it, to compress it.

6th. Has slept well from the time of application of band. Head diminishing.

7th. Continues to diminish. India rubber exchanged for a band of cotton.

From 7th to 24th, pressure continued; little change in head; remains stationary, at about 19 inches in circumference.

24th. Threw in ¾ of a dr. of solution; no effect.

Dec. 2d. Injected f. dr. jss. of solution. Head increasing; no effect.

15th. Head soft; diminished in size ½ inch in 24 hours.

At the first injection, but little fluid was allowed to escape. It was transparent, and showed, under the microscope, some epithelium cells. At the last operation, about one oz. was withdrawn; it was of a straw color; gave a slight cloud on application of heat, and was gelatinous on evaporation.

18th. Injected f. dr. jss. of the following solution: iodine, iij.; iod. pot., gr. vj.; aq. dist., oz. j.; no effect.

30th. Threw in f. dr. jss. of the following solution: iodine, grs. ivss.; iod. pot. gr. xij.; aq. dist. oz. j.; no effect.

1850; Jan. 5th. Head is increasing rapidly; measures 22 inches in circumference.

Injected gr. ij. iodine; grs. v. iod. pot. in f. dr. ij. dist. water. The fluid drawn off this time amounted to oz. iij.; was clear: exhibited, on standing, a filamentous appearance, like an exceedingly slight coagulum, and yielded, with starch, no trace of iodine.

9th. Child was restless, and head slightly hotter than usual, after the last injection.

12th. Child well; head flaccid. Drew off oz. xij. fluid, and injected the same quantity as before of the solution.

15th. Child slightly restless after injection, but soon became quiet, and exhibited no effect except a free secretion of tears and mucus from the eyes.

18th. Injected gr. iv. iodine, and iod. pot., gr. x. in f. dr. vj. water.

19th. No effect except the secretions from the eyes.

22d. Child in perfect health; appears unusually bright, and is very active, moving its hands actively, and its feet slightly. Tumor of back reduced to a level with the surrounding skin. Urine retained naturally, and the excoriations healed.

26th. Drew off oz. xvj. fluid, and injected gr. viij. iodine, and gr. xxiv. iod. pot., in water, f. oz. j.

Scarcely any effect was produced by this injection. The head was stationary for about four days after the injection, and then gradually increased. Compression by adhesive straps, bandages and gun cotton were tried perseveringly, without effect. Head 24 inches in circumference.

Feb. 4th. Drew off f. oz. xxiv., and injected gr. xij. iodine and gr. xxxvj. iod. pot. in oz. j. water. No effect was produced at the moment, but in the evening, 12 hours after the injection, there was twitching of the limbs and vomiting. The matter vomited colored the starched dress of the child of a dark color. The dress, diapers, bed, and handkerchief used on it were impregnated with the iodine odor for about three days after the injection, but the most careful tests, by Prof. Blaney, with starch, after adding chlorine water, never detected any trace of iodine in the fluid drawn from the head.

Feb. 19. For several days the head remained stationary, but has commenced to enlarge. Injected gr. x. iodine, with gr. xxx. iod. pot., after evacuating oz. xxiv. fluid. Head in this and other cases, when much fluid was drawn, supported with adhesive straps and bandages.

Effect the same as in the last instance, cold lotion was applied to the head, and purge of castor oil given.

March 5th. Head increasing. Drew off xxiv. oz. fluid, and injected the same quantity as before. Applied a laced cap to the head.

The symptoms resulting from this injection were but slight.

March 9. Drew off vj. oz. fluid, and repeated the same injection. This quantity reduced the head to the same size as before, by which it appears that 1½ oz. serum formed daily.

Remarks.—During the treatment for the last two months, the child has grown rapidly; the face, which was triangular and thin, has filled up, is fat and firm; the skin, from being pale, is become ruddy; the limbs are firm, and the skin of the head, from having been glistening, smooth, with but little hair, and large veins ramifying over it, is covered with a thick growth of hair, and appears very natural, the bones of the opposite sides touch, and the head is 20 inches in circumference; the bones being thick and firm. The tumor of back is entirely obliterated, and the skin in its former position becoming firm and of natural color.

10th. No effect; head paler than natural.

11th, 12th. Child restless.

15th. Is in good health. Drew off f. oz. vi., and injected gr. vij. iodine, with gr. xxj. iod. pot. in oz. j. of water.

20th. No effect from last injection; used gr. x. iodine, with thrice the quantity iod. pot. in the oz. water. Fluid drawn, oz. vj. A few bubbles of air probably passed in with the injection.

21st. Has had twitching. Head pale.

27th. Drew off the same quantity, and injected as before.

28th. Little effect; dress colored by matters vomited.

April 3d. Repeated the injection with gr. xij. iodine, and gr. xxxvj. iod. pot. to the f. oz. of water. It produced vomiting and purging of matters which colored the clothing. It is probable the materials used were not pure, as they were not procured from the usual shop, and had a different effect from any former injection. Since the first commencement of the treatment, it had been noticed that immediately after tapping, or while the head was diminishing in size, the child was wakeful, nervous, and affected with muscular twitching, on the slightest cause, while, on the contrary, it was quiet and had a tendency to sleep, when the head was full and increasing in size. Recently this tendency to somnolence has greatly increased—so much so that it could be at times scarcely roused. It was evident the disease was approaching its natural termination.

April 11. Threw in gr. ix. iodine, with gr. xxvij. iod. pot. in f. oz. j. dist. water, after withdrawing oz. vj. liquid. Slight twitching of the limbs the next day.

April 17. Repeated the same quantity, after withdrawing f. oz. viij. liquid.

After about 12 hours had elapsed, the child was affected with severe and dangerous symptoms, twitching, vomiting of matters which colored the clothing dark. These symptoms continued 48 hours, and were the worst the child had experienced since the commencement of the treatment.

The analogy supposed to exist between spina bifida and hydrocephalus had at first given rise to the expectation that the treatment of the latter disease by injections of iodine, would be found efficient in its cure. This hope, after a fair trial, had in this case been abandoned, but the treatment was still continued, in the hope that its obvious effect in retarding the increase of the head might enable the bones to close at a size which would allow of health and intelligence in the child.

The gradually increasing severity of the symptoms, following each injection, and the tendency to somnolence and diminished activity and intelligence of the child, suggested the propriety of suspending their use.

Accordingly, on the 2d May, I drew off viij. oz. fluid, without any injection, the tension of the head and somnolence rendering such relief necessary. For two or three days, this afforded relief, but fluid accumulated with unusual rapidity, so much so that during my absence, in attendance on the Medical Convention, at Cincinnati, the head attained a great size, the child became somnolent, and Professor Herrick was called on to tap the head, which he did, May 12th, to the extent of vj. oz., with but partial relief.

It was in this condition that the head was found on my return, May 14. It was greatly increased in size, hotter than usual, covered with large, branching veins, and the child somnolent.

Notwithstanding, the approaching fatal result of the case was apparent, yet the rapid aggravation of the symptoms under the treatment by tapping alone, as compared with that by tapping and injection, induced us to resort again to the latter, as a palliation; accordingly, on the 15th, I threw in gr. vj. iodine, with thrice that quantity iod. pot. in oz. j. water. The amount of fluid previously drawn off was oz. xij.

No perceptible effect was produced 12½ hours, when the child commenced vomiting and purging, fell into convulsions and died immediately.

*Examination after Death.*—The back, in the situation of the former tumor, was found smooth, firm, with but slight traces of disease. A cure of the spina bifida had evidently been effected.

On laying open the cranium, two-and-a-half pints of clear fluid was discharged. This was contained, principally, in the enlarged ventricles, there being but a thin layer on the surface. The arachnoid membrane was in a perfectly natural state, thin, polished, delicate; without any thickening or opacity. At the point where all the punctures but two had been made, the two layers of the arachnoid were closely adherent to each other; so that all the injections had been made into the ventricles, and not, as I had thought probable, into the sac of the arachnoid. The deception, in this respect, arose from the extreme tenuity of the ventricular walls, which over the upper part of the cerebrum were scarcely a line in thickness, and at points composed of the investing membranes of the organ, the cerebral substance having entirely given way, so that the canula could be easily felt, when in the ventricle, by the finger on the outside of the head.

Descending to the lower surface of the cerebrum, the walls of the cavity became thicker. The optic thalami, and corpora striata, although separated from each other, were entire, and, with the exception of flattening, in their natural state. The corpora quadrigemina, medulla oblongata, and cerebellum were well developed and healthy.

The internal surface of the immense cavity, was composed of white nervous tissue, firm and smooth, except at points where there were slight elevations, without any sign of lymph effused or unusual vascularity. Indeed, the whole substance of brain and its membranes, seemed less vascular than natural. The corpus callosum, extremely thin but not divided, formed the roof of the cavity. The *iter ad infundibulum* was dilated, but the aquaduct of Sylvius seemed not pervious without pressure. On the floor of the cavity lay the choroid plexus, hypertrophied, and resembling a congeries of mucous follicles, rather than the natural condition of the membrane.

Between the cerebrum and cerebellum was found a cyst of the size of a small plum, containing a serous fluid. On opening this, there was found a small surface, resembling entirely that of the choroid plexus in the large cavity.

This circumstance, together with the perfectly healthy appearance of the arachnoid, induced Professor Herrick, who assisted me in this examination, to believe that the common opinion, that *internal* chronic hydrocephalus originates from the arachnoid membrane, in so far, at least, as this case is concerned, is erroneous. He thinks the diseased choroid plexus was the source of the fluid. On examining that tissue with the microscope, his opinion (in which Professor Davis concurred) was confirmed, for he found in the tissue a great number of cells, arranged in groups, or in form of racemes attached to a common footstalk.

I am under the deepest obligation to Professor Herrick, for assistance during the treatment of this case. I was also assisted by Professors Evans, Blaney, Davis, Dr. David Rutter, and several other medical friends.

Notwithstanding the general preference in Europe of tincture iodine to the solution, and the success which appears to have followed its use in the hands of Dr. Tournesko, I adhere to my preference of Lugol's solution. This preference is founded upon what I regard as an established fact, that alcohol thrown into serous cavities is liable to produce violent inflammation, and upon the results of my own experience. I have used the solution with success in a very large number of cases of hydrocele, several of spina bifida, in empyema, in serous cysts, in dilated lymphatics, in ascites, in ovarian cyst. I do not use it at present either in hydrarthrosis or in ascites, but this does not arise from any accident which has happened with it in my own practice, but from the unfavorable results which are said to have followed the use of the tincture of iodine in these cases.

I regard chronic hydrocephalus as the most favorable form of dropsy for treatment by injections of iodine after hydrocele. The reasons are, that it is contained in a cyst, the internal surface of which is destitute of nerves, and consequently without sensibility, destitute in many cases of red vessels, and the irritation of which produces neither sensation nor spasm. In ascites, the surface of the peritoneum exposed to the action of a solution injected is so great, that danger may be apprehended. In hydrarthrosis the fluid effused is synovial, and the case is, therefore, less favorable for injections than serous cysts. There is a difficulty, however, in treating hydrocephalus not met with in other dropsies, which requires to be considered. It is, that the bones of the cranium to a certain extent prevent the walls of the sac from being brought together, and therefore prevent the discharge of the fluid injected. Now, this does not by any means contra indicate the performance of the operation. In hydrocele, and in many other cases, it is even deemed desirable to leave behind a certain part of the fluid injected. If the fluid cannot be withdrawn, a smaller quantity must be used. The degree of inflammation desired is not by any means high. Still I am of the opinion, that it will be found difficult to inject a sufficient quantity of iodine into the ventricles of the brain, to excite the requisite action of their surface, without leaving so much of it as may constitute an overdose when absorbed and carried by the circulation into the different organs.

The plan which I should recommend at present to avoid this difficulty is this: 1, Use a trochar of a certain size, as that employed for hydrocele; 2, compress the head, and draw off as much fluid as practicable; 3, inject a solution of iodine of such strength that, when mixed with the fluid remaining in the ventricles, it will still be of the strength of $\frac{1}{2}$ gr. of iodine to the oz. of liquid; 4, throw this through a gum elastic tube, carried through the canula into the ventricles; 5, inject the fluid first drawn off through the same tube to expel the solution.

If distilled water at the temperature of the body be found as harmless as the serum, which I see no reason to doubt, I should advise to inject it so as to expel all the solution introduced. In this case the solution might be made much stronger. If distilled water should be found objectionable, it is probable that an artificial serum might be used instead of it.

It might be expected, before concluding, that I should lay down some rules for selecting the cases which are proper for operation. It is obvious that in case of deficiency of the cerebrum an operation would be useless. It is equally plain, that where the disease has advanced to its latest stage, when there are frequent convulsions, or great derangement of the functions necessary to life, an operation would not be advisable. Beyond this I am not prepared to go at present, not having data upon which to base any rules which it might seem desirable to lay down.

I cannot but feel the need of sustaining my recommendation of this method of treatment by such authorities as may be favorable to it. M. Monod, in a report made to the Society of Surgery of Paris, March 16, 1854, on the subject of the case herein given, says, "it is to be regretted, in view of the innocence of the injections made by M. Brainard, that he had not tried them stronger and more abundantly;" and he adds, "this case, notwithstanding the want of success of the treatment, is well calculated to encourage new attempts in this direction."* It is needless to say, that I have shared the regret of M. Monod in this respect, but at the same time did not think it prudent to push the treatment further.

M. Boinet presented to the Society of Surgery, March 18, 1857, a report on the case of M. Tournesko, Surgeon of the Civil Hospital of Koltza, at Bucharest (Wallachia). The conclusion of this report is in these terms, "if the case of Mr. Brainard is not an example of complete cure, it proves at least with that of M. Tournesko, the innocence of iodine injections, which would have had, perhaps, a better success, if the important alterations of structure revealed at the autopsy had not existed. These two cases, and that of our *confrere* of Bucharest particularly, are not the less of a nature to encourage the use of these iodine injections in the cases where dropsy of the arachnoid does not extend to the ventricles of the brain, and where this organ is properly developed." "We think, then, that we may conclude with these facts only that iodine injections used with the necessary precautions are not only innocent, but efficacious, in the treatment of certain forms of hydrocephalus, when they are not accompanied with alterations of the cephalic substance." These reports did not, perhaps, receive the formal sanction of the learned society in all their details. No objections were, however, made, and the wise and conservative character of this, as of all similar bodies in France, where opinions are often taken as a warrant for practical action, is a guarantee that nothing rash or hasty would receive even an indirect sanction.

The individual authority of the reporters themselves is not trifling in this matter. Mr. Monod is a surgeon of Paris, of high standing, who has devoted especial attention to the subject

---

* Gazette des Hopitaux for 1854, p. 147.

of iodine injections, and treated by their means many difficult cases, some even of ovarian cyst, with success. M. Boinet is the distinguished author of an excellent work on *Iodotherapie* (therapeutical uses of iodine), which, although it is well known, is less so than it deserves to be, particularly in this country. He is not only thoroughly acquainted with the subject, but is a prudent and judicious surgeon of extensive experience.

This subject is brought forward at the present time for the purpose of inviting attention of surgeons to the advantages of this method of treating a disease generally incurable by other means. Many arguments in its favor have been omitted for fear of being too prolix. No other cases than those herein given are known to me in which this treatment has been used. It has never proved injurious. It is, therefore, hoped that the facts herein presented may be sufficiently conclusive to induce a trial of this plan of treatment first proposed, and put in practice in this country.

